# Analysis of the composition and antioxidant status of breast milk in women giving birth prematurely and on time

**DOI:** 10.1371/journal.pone.0255252

**Published:** 2021-07-23

**Authors:** Agnieszka Chrustek, Agnieszka Dombrowska-Pali, Dorota Olszewska-Słonina

**Affiliations:** 1 Department of Pathobiochemistry and Clinical Chemistry, Faculty of Pharmacy, L. Rydygier Collegium Medicum in Bydgoszcz, Nicolaus Copernicus University in Torun, Torun, Poland; 2 Department of Perinatology, Gynecology and Gynecologic Oncology, Faculty of Health Sciences, Collegium Medicum in Bydgoszcz, Nicolaus Copernicus University in Torun, Torun, Poland; Texas A&M University College Station, UNITED STATES

## Abstract

**Background:**

Breastfeeding with mother’s milk is the best form of nutrition not only for newborn babies, but especially for premature babies, due to the health benefits of taking human food.

**Objectives:**

The aim of the study was to examine the basic composition, cortisol concentration and antioxidant status of breast milk samples from women giving birth before 37 weeks of pregnancy and comparing it with milk samples from women giving birth after 37 weeks of pregnancy.

**Methods:**

The material for the study was milk taken from women giving birth before and after 37 weeks of pregnancy. The basic composition of breast milk was determined using a MIRIS analyzer, cortisol concentration in samples by an enzyme-linked immunosorbent test and their total antioxidant status was assessed by DPPH and FRAP methods.

**Results:**

It has been shown that the concentration of cortisol in samples of human milk in the group of women giving birth before 37 weeks was 13.95 ng / ml [4,71–86,84], while in the group of women giving birth after week 37 of pregnancy—10.31 ng / ml [2.35-39-02] (p = 0.014), while% inhibition of DPPH was 65.46% and 58.30%, respectively (p = 0.014).

**Conclusions:**

Milk from women giving birth prematurely is qualitatively different from the milk of women giving birth on time. The total antioxidant status of preterm milk is higher than term milk, which promotes the development of premature babies. Higher cortisol levels in samples from women giving birth before 37 weeks of pregnancy may stimulate the digestive system.

## 1. Introduction

WHO (World Health Organization) and UNICEF (United Nations International Children’s Emergency Fund) recommend exclusive breastfeeding up to the age of 6 months and continue feeding with complementary nutrition up to 2 years of age and longer if mother and child need it because of the health-promoting properties of human milk [1]. Breastfeeding protects a child against the occurrence or milder course of diseases such as sudden infant death syndrome, sepsis, necrotizing enterocolitis, gastrointestinal tract infections, respiratory tract infections, otitis media, bacterial meningitis, type I and type II diabetes, overweight, obesity, hypercholesterolemia or allergic diseases [2, 3]. This process can also protect the mother by reducing the risk of postpartum bleeding, accelerating uterine involution, increasing bone remineralisation up to pregnancy, and reducing the risk of ovarian and breast cancer [1, 4]. Human food ingredients, such as IgA antibodies, enzymes, hormones, growth factors and other immune factors may be responsible for the above mentioned benefits. Breast milk has many biological functions, e.g. anti-inflammatory, anti-infective and strengthens the immune system [5].

There are many hormones in breast milk, such as melatonin, thyroid hormones (thyroxine, triiodothyronine), thyrotropin, prolactin, fat hormones (resistin, adiponectin, leptin), parathyroid hormone or cortisol. Cortisol (glucocorticosteroid, stress hormone) produced by the adrenal cortex, affects metabolism, and also has anti-inflammatory properties. It is a very important component of breast milk due to the regulation of the digestive system and the impact on the child’s neurological development [6]. It is believed that cortisol concentration in breast milk may be related to infant temperament [7]. People with low levels of this hormone are more likely to get sick [8]. Researchers suggest that some premature babies have low serum cortisol [9].

According to the latest recommendations of the European Milk Bank Association (EMBA) Working Group (WG), food for a group of newborns with a very low body weight (less than 1500 g) is breast milk, supplemented if necessary with milk bank food and / or a breast milk booster containing protein hydrolyzate whey and casein, minerals (especially calcium and phosphorus), vitamins and trace elements, as well as increasing the energy value of breast milk [10]. Female milk is the preferred food source for feeding premature babies due to the modulation of the immune response, protective action against necrotic enteritis, and anti-infective effect [10, 11]. The milk produced by mothers who gave birth before the due date differs qualitatively and quantitatively from the milk of mothers who gave birth on time. These differences are most visible in the early stages of lactation (colostrum), but in later stages of lactation these differences decrease [12].

The aim of the study was to examine the basic composition, cortisol concentration and antioxidant status of breast milk samples from women giving birth before 37 weeks of pregnancy and comparing it with milk samples from women giving birth after 37 weeks of pregnancy. Our research suggests that the total antioxidant status of preterm milk will be higher compared to term milk due to the protection of premature babies against free radicals. Our research focused on mature milk, which is an additional advantage of the research and shows that over the course of lactation, the antioxidant status of breast milk of women giving birth prematurely remains at a high level. We also believe that cortisol levels in milk samples from premature births may be higher because of the stress that women experience.

## 2. Materials and methods

### 2.1. Participants

The experiment was approved by the Bioethics Committee of the Nicolaus Copernicus University in Toruń, Collegium Medicum in Bydgoszcz no. KB437/2018 and KB121/2019. The study included women from the Warmian-Masurian (n = 1), Pomeranian (n = 5) and Kuyavian-Pomeranian (n = 84) voivodships, residing both in the village and in the city, giving birth before 37 weeks of pregnancy (26.1 to 36.6 weeks)) 37 weeks of pregnancy (study group, n = 50) and after 37 weeks of pregnancy (37.0 to 40.0 weeks) (control group, n = 40). Participation in a research project was voluntary. The selection of the study participants was purposive. Before starting the study, the obstetricians were informed about the purpose and course of the research. The inclusion criteria for patients in the study included: feeding the baby’s breast milk or expressed breast milk using a bottle/probe, no history of depression and no anti-anxiety or anti-depressant therapy during of the last year, physical and mental condition allowing independent expressing breast milk and completing a survey questionnaire. Milk donors signed the consent and completed the questionnaire. The survey contained questions, about age, weight, place of residence, pregnancy, type of delivery, week of pregnancy. The women participating in the study did not take medications and did not suffer from chronic diseases. All breastfeeding women applied a healthy, balanced diet with no exclusions.

The average age of women giving birth before 37 weeks of gestation was 31±5 years, and their BMI was 25±1 kg/m2, and for women giving birth after 37 weeks of pregnancy 29±4 years and 24±3 kg/m2 respectively.

### 2.2. Samples of breast milk

Samples of breast milk came from 4–5 weeks of lactation (mature milk). It was important to conduct the study at this time due to the stabilization of lactation. Including in the study mothers of preterm infants shortly after giving birth could be challenging due to anxiety and discouragement with the initial small amounts of milk. The increase in the amount of breast milk due to the appropriate frequency of pumping (8–12 sessions) made it possible to analyze the milk of premature babies’ mothers. The donors were asked to donate about 40 ml of breast milk. Expressing milk with an electric breast pump. During the day, the studied sample of women expressed milk four times, in four time periods 06: 00–12: 00, 12: 00–18: 00, 18: 00–24: 00 and 24:00–06:00. For each time period, the desired amount of expressed milk is 10 ml, 5 ml before attaching the baby to the breast and 5 ml after finishing the feeding. Each batch of milk was poured into one collective bottle. Milk obtained from the daily collection of milk made it possible to analyze the nutritional composition of the milk and the caloric level. In order to determine the level of cortisol and antioxidant status in human milk, the participants of the study pumped a single portion of milk (20 ml) around 8.30 am. The breast milk was stored in the refrigerator for no longer than 24 hours.

Milk samples were frozen at -80° C for analysis, but not longer than 6 months. Prior to analyzing the antioxidant status and testing cortisol concentration in human milk, samples of breast milk were gradually thawed, centrifuged at 3000 x g for 15 minutes at 5°C. The defatted supernatant was pipetted and used for analysis.

### 2.3. Determination of the basic composition of breast milk

MIRIS Human Milk Analyser (Miris AB, Uppsala, Sweden)was used to analyze macronutrients [total fat (g/100mL), protein (g/100mL), total solids (g/100mL) and energy content (kcal/100ml)] in milk samples. Each sample before analysis was heated at 40°C in a thermostatic bath and then homogenized using MirisSonicator [1,5 sec/Ml]. Each sample was analyzed in triplicate.

### 2.4. Determination of cortisol concentration in human milk

For the determination of cortisol in breast milk, a commercial enzyme-linked enzyme test (DiaMetra, Italy) was performed. For analysis, 25 μl of breast milk samples and standards were applied to a 96-well plate. Ready-made reagents (standards, TMB, antigen, stop solution) included in the kit were used for the test. The result was read using a plate reader (MultiskanGo, ThermoScientific) at a wavelength of 450 nm. The cortisol concentration in each sample was determined in duplicate and the test was previously validated on human milk samples.

### 2.5. Determination of the antioxidant activity of breast milk using the DPPH •radical

#### 2.5.1. Reagents

DPPH (2,2-diphenyl-1-picrylhydrazl) (Sigma Aldrich, USA), methanol (Chempur, Poland), glacial acetic acid 99.5% (Chempur, Poland),

#### 2.5.2. Method

The method of Atanassov et al. 2011 with modifications was used in the work. A 100μM methanolic solution of DPPH was prepared before the experiment [13].

1 ml of the prepared DPPH solution was pipetted into Eppendorf-type tubes, 250 ml of breast milk was added, vortexed, and then incubated for 60 min in the dark at room temperature. Before reading, the samples were centrifuged for 2 minutes at 1500 x g, room temperature. Absorbance was measured at 517 nm against reference test (methanol). The control sample was a 100 μM DPPH solution.

The % inhibition was calculated from the formula:

$$\frac{absorbance\;of\;the\;control\;sample-average\;absorbance\;value\;of\;tested\;milk}{absorbance\;of\;the\;control\;sample}\times 100$$

### 2.6. Determination of the ability of breast milk to reduce Fe (III) ions

#### 2.6.1. Reagents

TPTZ (2,4,6-Tris- (2-pyridyl) -s-triazine) (Sigma-Aldrich, USA), hydrochloric acid (Chempur, Poland), iron (III) chloride 6 hydrate (Chempur, Poland), sodium acetate 3 hydrate (POCH, Poland), ascorbic acid (Chempur, Poland).

#### 2.6.2. Method

The work uses the FRAP (Ferric Reducing Antioxidant Power) method according to Benzie and Strain 1999 with modifications [14].

A reagent consisting of 300 mM acetate buffer pH 3.6 was used in the experiment; 10mM TPTZ in 40 mM hydrochloric acid and 20 Mm iron (III) chloride hydrated in a ratio of 10:1:1. The prepared reagent was the blank for the experiment.

The experiment consisted of using a 50 μl breast milk sample and 1.5 ml prepared reagent, vortexing, then reading the absorbance at 0 minutes of the experiment at 593 nm. The sample was incubated at 37°C for 4 min, then vortexed and absorbance at the same wavelength was measured.

A calibration curve was prepared using 6 ascorbic acid solutions with concentrations: 100, 200, 400, 600, 800, 1000 μM. The calibration curve showed the dependence of the absorbance of ascorbic acid on its concentration.

The FRAP value was calculated using the formula:

$$\frac{change\;in\;human\;milk\;absorbance\;from\;0\;to\;4\;minutes}{change\;in\;absorbance\;of\;the\;standard\;from\;0\;to\;4\;minutes}\times standard\;concentration\times 2$$

* 2—factor using ascorbic acid

### 2.7. Statistical analysis

For statistical analysis, the Statistica 13.1 software package from StatSoft® was used. The normality distribution was verified by the Shapiro-Wilk test. There was no normality in the distribution of quantitative variables analyzed. Parameter variability is presented in the form of median, minimum and maximum values (min-max), and interquartile range (IQR). In order to assess the strength of the relationship between the studied parameters, the Spearman rank correlation test was used. The results at the level of p<0.05 were considered statistically significant. The non-parametric U Mann-Whitney paired test was used to assess statistical significance in two groups of independent variables without normality of distribution.

## 3. Results

### 3.1. The basic composition of breast milk

In the presented studies, the basic composition of milk of women giving birth before 37 weeks of pregnancy compared to the basic composition of milk of women giving birth after 37 weeks of pregnancy did not differ statistically (Table 1).

**Table 1:** Basic composition of breast milk from women giving birth before and after 37 weeks of pregnancy.

Parameter	Samples of breast milk from women giving birth before 37 weeks of pregnancy (n = 50)			Samples of breast milk from women giving birth after 37 weeks of pregnancy (n = 40)			p
	Me/IQR	Min	Max	Me/IQR	Min	Max	
**Fat [g/100ml]**	3.60/1,10	2.00	6.20	3.70/1.70	1.30	6.80	0,856
**Total protein [g/100ml]**	1.50/0.30	1.10	5.00	1.50/0.20	0.90	2.40	0,127
**Nutritional protein [g/100ml]**	1.20/0.30	0.90	4.00	1.20/0.20	0.70	1.90	0,242
**Carbohydrates [g/100ml]**	7.85/0.80	4.20	8.70	8.10/0.50	3.30	8.80	0,149
**Dry matter [g/100ml]**	13.30/1.50	1.30	16.80	13.50/1.40	6.90	16.80	0,338
**Caloricity [kcal/100ml]**	74.00/12.00	50.00	100.00	74.00/16.00	50.00	100.00	0,780

Positive correlations were found for parameters of breast milk samples from women giving birth before 37 weeks of pregnancy (Table 2). among others between age and the concentration of carbohydrates, fat and dry matter, the amount of fat and caloricity, the level of total protein and nutritional protein, the amount of dry matter and the concentration of carbohydrates.

**Table 2:** Correlations of Spearman rank order significant with p <0.05 in a group of milk samples from women giving birth before 37 weeks of pregnancy (n = 50).

Variable	r	p
age & carbohydrates	0.308	0.029
fat & dry matter	0.829	<0.001
fat & caloricity	0.976	<0.001
total protein & nutritional protein	0.974	<0.001
total protein & dry matter	0.307	0.029
carbohydrates & dry matter	0.423	0.002
dry matter & nutritional protein	0.285	0.044
dry matter & caloricity	0.853	<0.001

### 3.2. Determination of cortisol concentration in human milk

**Fig 1:**
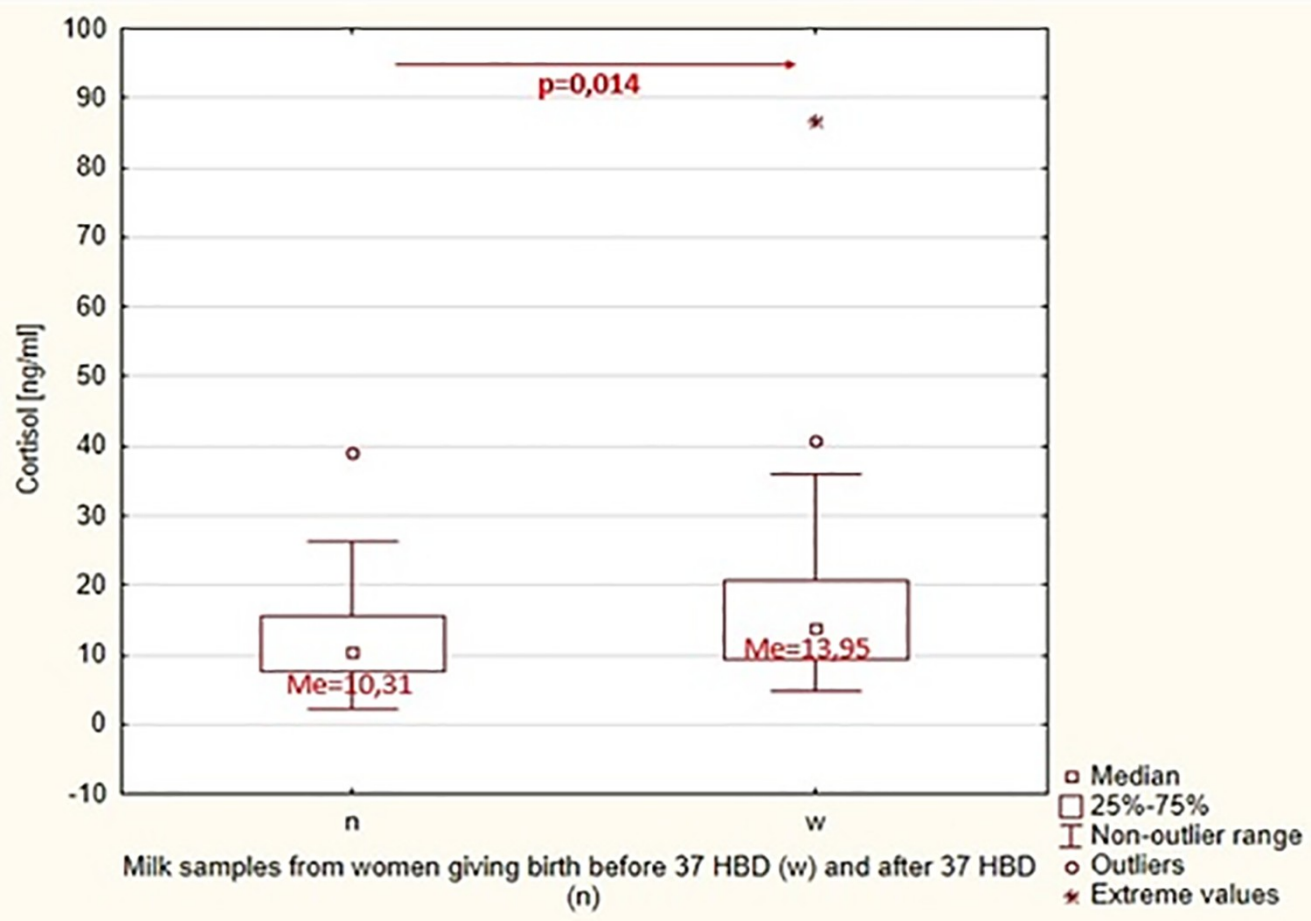
Statistical significance of differences in cortisol concentration in breast milk between the group of women giving birth before and the group of women giving birth after 37 weeks of pregnancy. n-milk samples from women giving birth after 37 HBD, w-milk samples from women giving birth before 37 HBD.

**Fig 2:**
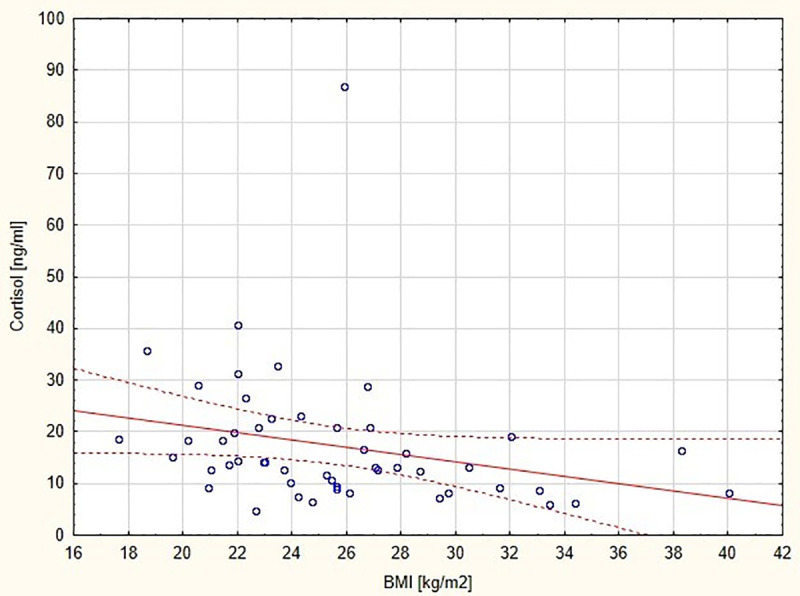
Graphic representation of the correlation between cortisol concentration in milk samples from women giving birth before 37 weeks of pregnancy and the mother’s BMI (y = 35.308–0.701x, r = -0.419, p = 0.002).

Our research showed that cortisol concentration in breast milk samples in the group of women giving birth before 37 weeks of pregnancy was 13.95 ng/ml [4.71–86.84], while in the group of women giving birth after 37 weeks of pregnancy 10.31 ng/ml [2.35–39.02]. There were statistically significant differences between the values of this parameter in the examined groups (p = 0.014) (Fig 1). In our studies, was observed negative correlation between the BMI of nursing mothers and the concentration of cortisol in natural diet (Fig 2).

### 3.3. Total antioxidant status of breast milk

**Fig 3:**
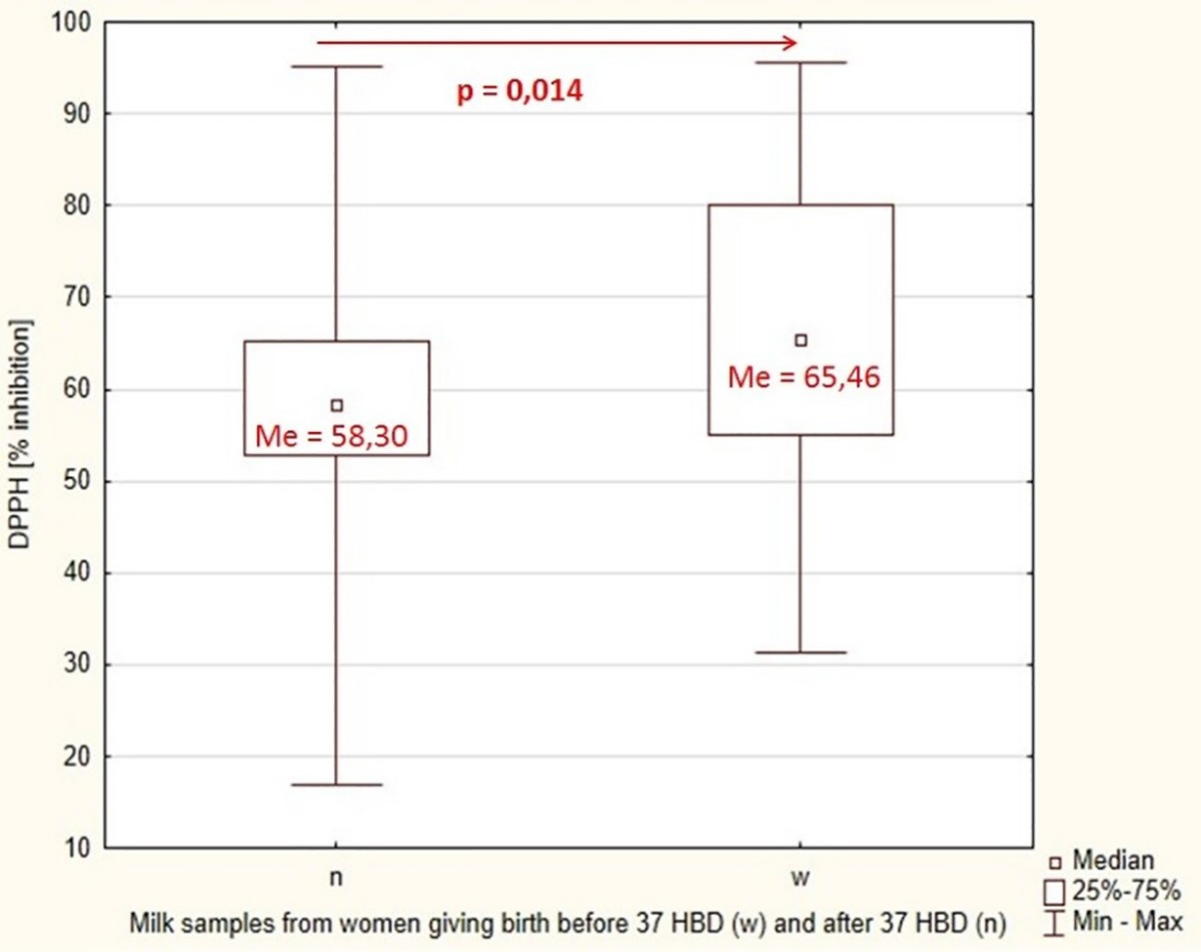
Statistical significance of differences in % DPPH inhibition in breast milk between the group of women giving birth before 37 weeks of pregnancy and the group of women giving birth after 37 weeks of pregnancy.

In the presented studies, in breast milk samples from women giving birth before 37 weeks of pregnancy DPPH inhibition was shown at 65.46%, while the total antioxidant status determined by the FRAP method was 552.61 μM. In the group of women giving birth after 37 weeks of pregnancy, DPPH inhibition of breast milk was 58.30%, and the total antioxidant status by FRAP was 760.79 μM. Table 3 presents the results of the assessment of the antioxidant status of breast milk made by the two methods in both groups. An increase in antioxidant status was observed in samples from prematurely giving birth compared with samples from women giving birth on time (for DPPH method p = 0.014) (Fig 3)).

**Table 3:** Results of the total antioxidant status of breast milk samples in the study groups.

Variable	Milk samples from women giving birth before 37 weeks of pregnancy (n = 50) Me [Min-Max]	Milk samples from women giving birth after 37 weeks of pregnancy (n = 40) Me [Min-Max]	p
**DPPH [% inhibicji]**	65.46 [31.40–95.50]	58.30 [16.89–95.10]	0.014
**FRAP [μM]**	552.61 [105.98–2134.75]	760.79 [127.17–14512.49]	0.476

## 4. Discussion

Mother’s milk improves the baby’s growth and neurological development, and reduces the risk of necrotizing enteritis and sepsis, which is why it should be the basis of nutrition for premature babies. The composition of preterm milk is different from the term milk. Scientific research indicates that milk from women giving birth before 37 weeks of pregnancy differs from the milk of women giving birth on time [15]. Results different from ours were presented by Bauer and Gerss [15]. Researchers analyzed breast milk from 1 to 8 weeks of lactation from preterm delivery women and compared them to the breast milk of women giving birth on time. They showed an increase in the amount of protein, carbohydrates, fats and caloricity in premature milk samples compared to term milk samples [14]. It is believed that premature babies’ milk contains about 1.8–2.4 g/100ml of protein in the first month of lactation, while milk from women giving birth at a time of 0.8–1.6 g/100ml. The differences in composition can be partly explained by the reduced volume of milk produced by premature births. An increase in protein concentration in milk is also a response of the infant’s body to the need for protein [16].

According to analyzes in breast milk from premature births, the fat content is estimated at 1.6–5.5 g/100ml, carbohydrates 2.2–7.5 g/100ml, and its caloricity is 61–92 kcal/100ml, while in breast milk taken from women giving birth at a time of 1.6–5.1 g/100ml, 3.0–7.2 g/100ml, 48–85 kcal/100ml respectively [17–19].

Groer et al. [18] observed a similar relationship in cortisol concentration in human milk to our study. Researchers collected human milk in the first week of lactation and measured the levels of cortisol and secretory immunoglobulin A (sIgA) in them. The concentration of sIgA and cortisol was higher in breast milk from premature births. In preterm milk, cortisol was 61.2 μg/dl, whereas in term milk 58.8 μg/dl. The study suggests that cortisol present in human milk may potentially affect sIgA secretion [18]. Higher levels of cortisol in the breast milk of premature women may be caused by stress, but it is worth noting that cortisol in these concentrations may be useful for protecting the premature baby’s body, stimulate the digestive system, and affect the neurological development of the newborn [6, 18]. In our studies, extreme and outlier values of cortisol concentration were observed in human milk samples of women giving birth at 26.1 and 31.0 weeks of pregnancy, respectively. It could be related with the stress that these women experience.

Different results were presented by Voorn et al. [9], who examined the concentration of cortisol and cortisone in breast milk in relation to the daily rhythm of maternal adrenal cortex activity, weekly in the first month of lactation. A reduction of cortisol and cortisone levels by up to 50% in preterm milk compared to term milk was observed. Studies suggest that glucocorticoid levels in breast milk correspond to the daily rhythm of the activity of the hypothalamic-pituitary-adrenal axis and are lower in mothers who give birth to premature babies [9]. It is believed that the reduction in the concentration of the aforementioned hormones in the milk of premature births can be caused by the maturation of the mammary gland, as well as the content of fat and protein in breast milk [20]. In our studies, no correlation was observed between cortisol levels and the basic composition of breast milk, while a negative correlation was found between the BMI of nursing mothers and the concentration of cortisol in natural diet.

Pundir et al. [21] examined milk from women giving birth between 28 and 32 weeks of pregnancy. Milk taken from premature mothers had more cortisone (4.48 [1.73] ng/ml) than cortisol (1.88 [1.34] ng/ml), with a strong correlation between both hormones (p = 0.001, r = 0.85). Cortisone was significantly higher in the milk of mothers who gave birth to infants after 30 weeks, compared to those who gave birth before 30 weeks of pregnancy (p = 0.02). Cortisone is a substance that can be converted into a potent hormone (cortisol), which suggests that cortisol levels may increase in later lactation. Glucocorticoid levels did not change during sampling (1–6 week lactation) [21]. Currently, there is little knowledge about the effects of glucocorticoids on the health and development of a breast-fed child, which is why further research on this topic is important.

It has been proven that breast milk, consisting of many biologically active ingredients such as enzymes, vitamins and proteins, is a powerful antioxidant that protects a child against free radical diseases. The consequences of the imbalance between antioxidant capacity and oxidative stress may be necrotizing enterocolitis, retinopathy, and many other disorders that may occur in premature babies. In order to provide maximum protection for the body and prevent oxidative-antioxidant imbalances, it is important to provide the child with antioxidants in the form of breast milk [22].

Similar results to ours were presented by Abuhandan et al. [23], in which the TAS (total antioxidant status measured in μmol H2O2) of premature mothers’ milk was also higher and was 0.21±0.03 compared with milk of mothers giving birth at a time (0.18±0.02) (22). Paduraru et al. [24] did not show statistical significance between samples in total antioxidant status tested by the ABTS method in breast milk samples in similar test groups [24]. Some data, like ours, show that the milk of premature women has more antioxidants compared to the milk of women giving birth on time [25].

On the other hand, other studies show a lower overall antioxidant status of preterm milk compared to term milk [26]. Quiles et al. [26] showed that the average total antioxidant status of colostrum, transitional milk, and mature milk in premature milk samples using the ABTS method is over 9 mM, respectively; 7 mM; 7 mM, while in term milk samples suitable over: 11 mM; 9 mM; 7 mM. The total antioxidant status of breast milk appears to be higher for colostrum compared to mature milk, and its radical scavenging activity decreases during lactation [26]. The discrepancy in results may be due to maternal factors, supplementation, perinatal factors, and eating habits of the nursing mother [25]. Scientists believe that the reduction of antioxidant levels in breast milk may be affected by stress, long hospitalization and mother’s health [23, 24, 27].

Carotenoids are an important group of antioxidants that have been tested in breast milk by Xavier et al. [28]. In the colostrum of women giving birth prematurely, a lower total carotenoids content was found compared to milk samples of pregnant women giving birth on time. Premature termination of pregnancy affects the quantitative composition of colostrum carotenoids, but has no effect on lutein content. This fact can be associated with the significant role of this xanthophyll in retinal development and cognitive function in newborns [28]. Giuliano et al. [29] showed that the content of carotenoids can change daily and even during feeding. The high variability of carotenoids may result from inter-individual differences, from the diet of a nursing mother, as well as from the bioavailability of carotenoids [29]. Antioxidative enzymes (e.g. catalase (CAT), superoxide dismutase (SOD), glutathione peroxidase (GPx)) are one of the most important elements of regulating the oxidation-antioxidant balance. In the milk of mothers giving birth on time, the activity of catalase ranges from 0.43 to 0.84 U/mg of protein, and in the milk of women giving birth prematurely—0.5–0.97 U/mg of protein [30]. L’Abbe and Friel (27) analyzed the levels of enzymes in preterm milk and in term milk from 1 to 12 weeks of lactation. They showed less SOD and GPx activity in milk than prematurely giving birth. Researchers believe that reduced food production in premature births may have an effect on the reduced content of antioxidant enzymes, resulting in a reduction in the antioxidant protection of breast milk. The concentration of antioxidant enzymes may also change depending on lactation phases, maternal and perinatal factors [27].

The literature on the subject shows that scientists around the world are constantly analyzing the composition of human milk. The conducted research provides many new insights into the properties and composition of breast milk. However, due to the uniqueness of breast milk, it is necessary to conduct further research in this area. Most research studies focus on colostrum or transitional milk, but we decided to investigate the variability of the composition of mature milk depending on the date of delivery. Our results indicate that mature milk (4–5 weeks of lactation) of women giving birth prematurely may have a higher concentration of cortisol as well as higher protection against free radicals compared to milk of women giving birth on time. We believe that the results of the conducted study are very important. However, the literature data on the concentration of cortisol in breast milk and the antioxidant protection of breast milk are contradictory, therefore it is worth expanding the study in the future to include the division of the group into milk from women who gave birth to extreme premature babies, premature babies and on time.

## 5. Conclusions

Premature mothers’ milk contains higher levels of cortisol and has a higher overall level of antioxidants compared to the milk of women giving birth on time. Changes in the hormonal economy may be caused by the stress of prematurely born women, but it is worth noting that cortisol in the presented concentrations (4.71–86.84 ng/ml) can regulate the digestive system and affect the neurological development of premature babies. The higher total antioxidant status of natural foods protects newborns against free radicals, which in turn can prevent the occurrence of necrotising enterocolitis or retinopathy in premature babies.
